# A Mobile App–Based Intervention (Parentbot–a Digital Healthcare Assistant) for Parents: Secondary Analysis of a Randomized Controlled Trial

**DOI:** 10.2196/64882

**Published:** 2025-04-17

**Authors:** Joelle Yan Xin Chua, Mahesh Choolani, Cornelia Yin Ing Chee, Huso Yi, Joan Gabrielle Lalor, Yap Seng Chong, Shefaly Shorey

**Affiliations:** 1 Alice Lee Centre for Nursing Studies, Yong Loo Lin School of Medicine, National University of Singapore Singapore Singapore; 2 Department of Obstetrics and Gynaecology, National University Hospital Singapore Singapore; 3 Department of Psychological Medicine, National University Hospital Singapore Singapore; 4 Saw Swee Hock School of Public Health, National University of Singapore Singapore Singapore; 5 School of Nursing and Midwifery, Trinity College Dublin Dublin Ireland; 6 Institute for Human Development and Potential, Agency for Science, Technology and Research (A*STAR) Singapore Singapore; 7 Yong Loo Lin School of Medicine, National University of Singapore Singapore Singapore; 8 Department of Obstetrics & Gynaecology, National University Health System Singapore Singapore

**Keywords:** perinatal, mobile app, app use, survey response, parents, randomized controlled trial, artificial intelligence, AI

## Abstract

**Background:**

Mobile app–based interventions are viable methods of delivering perinatal care support to parents. A mobile app–based intervention entitled Parentbot–a Digital Healthcare Assistant (PDA) was developed and evaluated via a randomized controlled trial. PDA aimed to provide informational, socioemotional, and psychological support to parents across the perinatal period. As developing such interventions is resource intensive, it is important to evaluate participants’ use and the components that are appreciated by them.

**Objective:**

This study aims to examine the (1) relationship between participants’ characteristics and PDA use, (2) relationship between PDA use and parenting outcomes, and (3) relationship between participants’ characteristics and the time taken to respond to the surveys (survey response timing).

**Methods:**

This study is the secondary analysis of a randomized controlled trial. A convenient sample of 118 heterosexual couples (236 participants: n=118, 50% mothers and n=118, 50% fathers) from a public tertiary hospital in Singapore were recruited. Data were collected from November 2022 to August 2023. Descriptive statistics were used to summarize the parents’ characteristics and study variables. Linear mixed models were used to examine the effect of (1) participants’ sociodemographic characteristics on PDA use metrics, (2) use metrics on parenting outcomes, and (3) participants’ sociodemographic characteristics on the survey response timing. The Pearson correlation was also used to examine the linear relationships between the PDA use metrics and parenting outcomes.

**Results:**

The following parental characteristics were found to be associated with PDA use: antenatal course attendance, gender, religion, ethnicity, and the number of children. After adjusting for baseline values and sociodemographic covariates, only the viewing of educational materials was statistically significantly associated with improvements in parents’ anxiety (β=–0.48, 95% CI –0.94 to –0.009; *P*=.046), parent-child bonding (β=–0.10, 95% CI –0.19 to –0.01; *P*=.03), social support (β=0.31, 95% CI 0.08-0.54; *P*=.01), and parenting satisfaction (β=0.57, 95% CI 0.07-1.07; *P*=.03) at 1 month post partum. Moreover, parents’ age, ethnicity, grouping, and number of children were found to be related to the survey response timing.

**Conclusions:**

As the viewing of PDA’s educational materials was linked to improvements in parents’ perinatal well-being, the provision of educational resources should be prioritized in future app-based parenting interventions. Because the use of other PDA features, such as poster activities, forum posts, and reflection and gratitude exercises, had a limited effect in improving parents’ well-being, future interventions could explore alternative activities to better engage parents. Future mobile app–based parenting interventions could conduct similar evaluations on app use and the effectiveness of specific features to validate the findings of this study.

## Introduction

### Background

Mental health issues, such as depression and anxiety, affect around 26% and 15% of mothers [1,2] and 8%-14% and 11% of fathers [3,4], respectively, during the perinatal period (from conception to 1 year post partum) [5]. Poor psychological health among parents can cause long-term physical, psychosocial, and emotional developmental problems among their children [6,7]. Singapore has recently declared perinatal depression among mothers as a public health concern [8]. Fathers in Singapore have also reported signs of perinatal stress and anxiety [9]. Hence, parents in Singapore require support during the perinatal period.

Mobile health apps have been found to provide timely and customized support to people with various medical conditions, such as depression, anxiety, and hypertension [10,11]. A recent review showed that mobile app–based interventions are very helpful for parents as they can receive valuable educational, socioemotional, and psychological support anytime and anywhere during the perinatal period [12]. As most of Singapore’s population consists of smartphone users [13], it is viable to conduct a mobile app–based intervention for local parents.

Participants’ attendance and adherence to the intervention components are necessary for the intervention to be successful. For in-person interventions, such data on attendance and adherence could be easily recorded and examined [14]. However, for mobile app–based interventions, where study participants have unlimited access to the intervention components, the examination of intervention dosage, including participants’ use of the app, becomes very crucial [15]. Thus far, participants’ use has been measured by varied methods, such as tracking the number of log-ins, number of activities completed, and the duration spent using the app [15,16]. As increased intervention use has been associated with better trial outcomes, previous studies that conducted technology-based interventions for various conditions, such as depression, psychological stress in people who are overweight, and for parents of children with externalizing behavior disorders, have analyzed dose-response relationships between intervention use and participants’ health outcomes [15-18]. These additional dose-response analyses could potentially help to pinpoint the optimum intervention use for participants to gain maximum benefit in the future [15-18]. Moreover, previous studies have reported that sociodemographic factors, such as age, gender, income, education level, and marriage status, could influence one’s intervention use [19-21]. Because the examination of participants’ intervention use could reveal valuable insights to inform future research, studies conducting mobile app–based interventions are encouraged to conduct such analyses.

With the rise in digital interventions, web-based data collection methods for research studies have also increased in popularity [22]. While web-based surveys are cost-effective and efficient [22], studies have reported poor response rates for them compared to other survey modes (eg, mail, telephone, and in-person) [23]. Researchers who use web-based surveys have used various methods to send the survey links, including email, text messages, or WhatsApp (Meta Platforms Inc) messages for participants to complete the surveys at their convenience [22,24]. However, at times, these have resulted in delayed survey responses, which could delay the overall data collection process. Inaccuracies in measurement could also occur when participants complete their surveys late (eg, 1-month postpartum measurement is completed at 2 months post partum), and this could affect the study’s internal reliability and validity [25]. Because sociodemographic factors, such as age, gender, ethnicity, and income, can influence one’s willingness to complete surveys [22,26,27], the trends of time taken to complete research study surveys need to be explored.

In Singapore, previous trials conducted using mobile app–based interventions reported that such interventions were feasible, promising, and helpful in supporting parents and improving parenting outcomes across the perinatal period [24,28,29]. However, parents’ use of these mobile apps was never explored [24,28,29], leaving researchers with little insight into the relationships between parents’ sociodemographic characteristics and app use and a limited understanding of which intervention components could be more helpful. Moreover, while a previous local study on parents collected data using web-based surveys [24], it did not examine the trends related to the time taken to complete the trial’s surveys. Therefore, this study seeks to address this research gap by investigating the app use and time taken to complete surveys related to a trial that evaluated the effectiveness of a mobile app–based parenting intervention.

### Project Overview, Aims, and Hypotheses

This study is a secondary analysis of a randomized controlled trial (RCT) that aimed to evaluate the effectiveness of a mobile app–based intervention entitled Parentbot–a Digital Healthcare Assistant (PDA) on improving parenting self-efficacy (primary outcome) and stress, depression, anxiety, social support, parent-child bonding, and parenting satisfaction (secondary outcomes) among parents until 3 months post partum. The parents were randomized to the control group receiving the standard perinatal care offered by the hospital and the intervention group receiving unrestricted access to PDA in addition to standard care. The results of the RCT are reported in another study [30]. This study aimed to examine the PDA use among the intervention group and the time taken to respond to the surveys (survey response timing) among both groups. The specific objectives were to examine (1) the relationship between participants’ characteristics and PDA use, (2) the relationship between PDA use and parenting outcomes, and (3) the relationship between participants’ characteristics and survey response timing. The study hypothesized that there were significant relationships between participants’ characteristics and PDA use as well as between participants’ characteristics and survey response timing. An increase in PDA use was also hypothesized to be related to a significant improvement in parenting outcomes.

## Methods

### Study Design and Participants

The RCT (trial registration: ClinicalTrials.gov NCT05463926) was conducted from November 2022 to August 2023. The secondary analysis of the RCT was conducted, and the findings were reported according to the STROBE (Strengthening the Reporting of Observational Studies in Epidemiology) guidelines [31]. A convenient sample of 118 heterosexual couples (236 participants: n=118, 50% mothers and n=118, 50% fathers) was recruited from the maternity clinics of a public tertiary hospital in Singapore. The primary researcher (JYXC) approached couples individually during their regular antenatal visits to the maternity clinics. Couples were given an overview of the study, and those who expressed interest were assessed for eligibility before providing their written informed consent. Heterosexual couples were included if they (1) were adults aged ≥21 years, (2) were fluent in English, (3) owned a smartphone with internet access, (4) planned to stay in Singapore until 3 months post partum, and (5) were having a low-risk singleton or multiple pregnancies at >24 gestational weeks (age of viability in Singapore) [32]. Couples who were expecting their first child and couples with existing children were included. Further details on the data collection process and eligibility criteria can be found in the published RCT study [30].

In this study, only parents in the intervention group were included in the PDA use analysis, and parents from both the intervention and control groups were included in the survey response timing analysis.

### Sample Size Calculation

The optimum sample size of 118 couples (n=59, 50% couples in the intervention group and n=59, 50% couples in the control group) was calculated based on assuming a medium effect size of ANOVA, *F* test value of 0.3, a power of 80%, a 2-sided 5% level of significance, and an estimated 30% attrition based on previous local trials [24,28,29]. This calculation was carried out using the PASS software (NCSS) based on a 1-way ANOVA, and further details can be found in the published RCT study [30].

### PDA Intervention

#### Overview

Parents in the intervention group were given unlimited access to PDA from >24 gestational weeks until 1 month post partum. During study recruitment, parents were given instructions to download PDA onto their smartphones, and each parent received a unique username and password to log in. A standardized video was used by the primary researcher (JYXC) to educate each couple on how to use PDA. PDA and its contents remained constant during the entire study.

**Figure 1:**
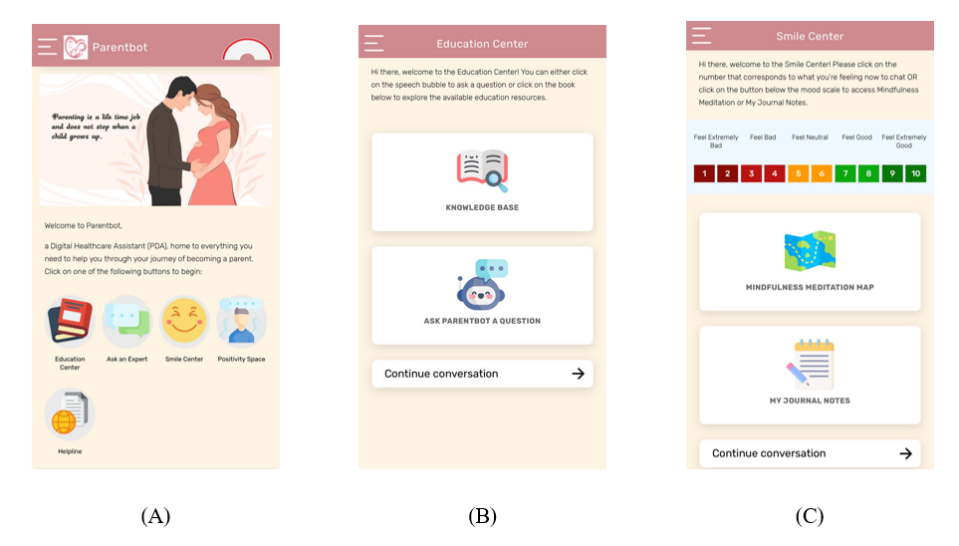
Screenshots of the Parentbot–a Digital Healthcare Assistant (A) home page, (B) education center, and (C) smile center.

An interdisciplinary team of researchers consisting of obstetricians, nurses, midwives, and technical experts worked together to develop PDA. PDA provided parents with informational, socioemotional, and psychological support using 6 components (Table 1). The education center provided parents with multimedia educational resources that were prepared by local health care experts (eg, midwives, obstetricians, and psychiatrists) and researchers. A rule-based chatbot (Parentbot) was present within this section to answer parents’ queries in real time. When Parentbot could not sufficiently answer any question, parents could choose the option of transferring their questions to the ask an expert platform for health care professionals (eg, midwives and nurses) to answer. The smile center provided parents with guided gratitude and reflection journal exercises and mindfulness-based meditation videos. In the positivity space, parents could interact with each other on the discussion forum, make simple digital posters containing positive messages, and view posters made by all parents on a digital board. PDA also had gamification features in the form of web-based badges (rainbow badge and helping hand badge) and slideshows. Parents were instructed to complete a set of tasks each week, with their progress being monitored and displayed in 7 sections to earn a rainbow badge. In addition, parents who posted, replied to, or commented on the discussion forum at least once a week would earn an extra web-based helping hand badge. Badges would be awarded to parents through 2 brief inspirational slideshows, 1 after childbirth and another at 1 month post partum. Finally, the helpline provided PDA user guides for parents to view, the technical team’s contact information, and information on appropriate local resources (eg, contacts of local public hospitals and social support services) for parents to seek help if needed. Screenshots of a few main features of PDA are presented in Figure 1.

**Table 1:** Details of Parentbot–a Digital Healthcare Assistant (PDA) features, corresponding theory, framework, or concept, and study outcome.

Name of the component	Contents	Theory, framework, or concept	Study outcome
Education center	Multimedia educational resources (readings, texts, audio files, pictures, and videos) on maternal, paternal, and infant care Embedded chatbot called Parentbot to answer questions in real time Parents can choose to transfer unanswered questions to the ask an expert platform	Self-efficacy theory by bandura [33]	Parenting self-efficacy
Ask an expert	Experienced nurses and midwives will respond to queries within 24 hours	Self-efficacy theory by Bandura [33]	Parenting self-efficacy
Smile center	A mood rating scale with differentiated chatbot responses to encourage parents to engage in the following activities: articles on emotional health, guided gratitude journal exercise, guided reflection journal exercise, and guided mindfulness-based meditation videos	Psychoeducation Positive psychology Mindful coping model	Psychological well-being
Positivity space	Discussion forum for participants to interact (support forum) Platform to make simple digital posters containing positive messages only (Poster-making Station) Digital board to view and like posters (Board of Hope)	Peer support Bandura self-efficacy theory Positive psychology	Social support Parenting self-efficacy Psychological well-being
Gamification features	Web-based badges (awarded based on weekly app activity) Two web-based slideshows given after childbirth and 1-month post partum	Self-determination theory	Participants’ engagement and motivation
Helpline	PDA use guides (text and video) Contact details of the technical team Contacts of useful local resources (contacts of local public hospitals, social services, and support groups)	—^a^	—

^a^Not applicable.

PDA’s features were developed using various relevant theories, frameworks, or concepts, and each feature was aimed at addressing different study outcomes as outlined in Table 1 and elaborated subsequently. More details on PDA’s features and development process can be found in a published developmental study [34].

#### Self-Efficacy Theory and Peer Support

The self-efficacy theory by Bandura [33] suggests that providing educational materials (texts, videos, audio, chatbot, and expert advice) could help parents practice and improve their baby care skills (mastery experiences) and receive valuable advice (verbal persuasion) to improve their parenting self-efficacy. The discussion forum also enabled parents to gain advice and learn from peers’ experiences (vicarious experiences) to increase their parenting self-efficacy [33]. Moreover, parents’ perceived level of social support could increase when they receive genuine empathy and invaluable realistic advice from their peers via the discussion forum [35,36].

#### Psychoeducation and Positive Psychology

Psychoeducation could help parents gain a clearer understanding of the symptoms, diagnosis, prognosis, and treatment of their psychological condition [37]. The gratitude exercises and web-based motivational posters were grounded in positive psychology principles, which focused on fostering positive emotions, behaviors, and thoughts rather than merely addressing negative thoughts and maladaptive behaviors [38]. Hence, psychoeducation and positive psychology interventions could enhance parents’ psychological outcomes [29,39].

#### Mindful Coping Model

The mindful coping model suggests that practicing mindfulness could help parents to positively reappraise their stressors by viewing previously threatening situations as beneficial [40], thereby helping them to manage their perinatal-related stressors [41] and improve their psychological well-being. To encourage positive reappraisal, parents would be guided to engage in reflection journaling exercises with Socratic questions (eg, “What can you learn from this situation?”) after mindfulness practices once they have reached a state of mindful awareness [42].

#### Self-Determination Theory

**Figure 2:**
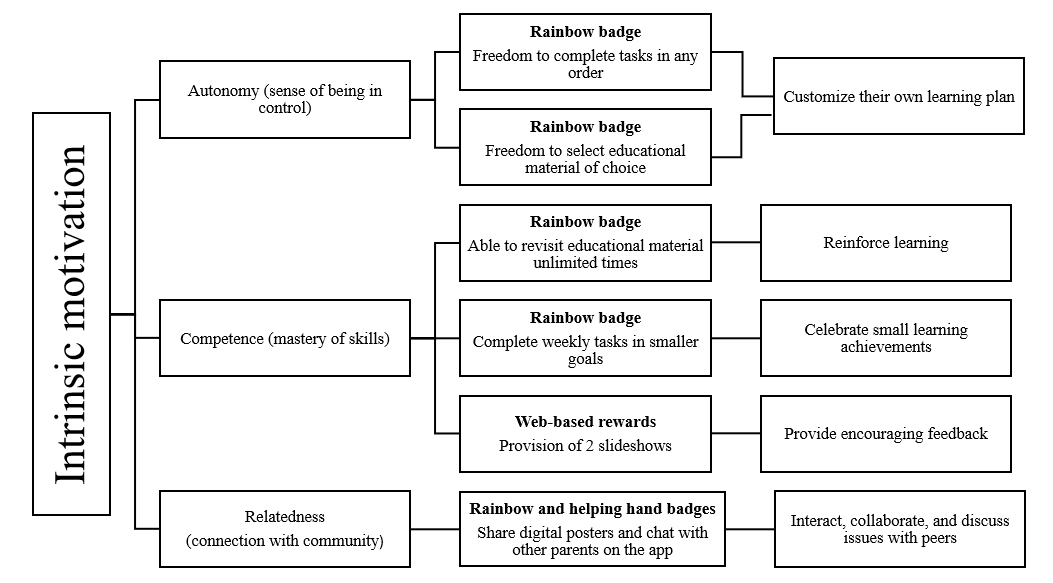
Parentbot–a Digital Healthcare Assistant gamification features and components of intrinsic motivation.

Gamification features were designed to boost parents’ motivation and engagement with PDA by addressing the following key components of intrinsic motivation outlined in self-determination theory: a sense of control (autonomy), confidence in skill mastery (competence), and connection with peers in the same community (relatedness) [43]. Figure 2 illustrates how the web-based badges and rewards support these components.

### Parenting Outcome Measurements

Parents’ sociodemographic childbirth-related details and parenting outcome measurements were collected using self-reported web-based surveys hosted on a secure, ethically approved server. Data were collected at 3 time points: baseline (>24 gestational weeks, age of viability in Singapore), 1 month post partum, and 3 months post partum. The survey links were sent to participants using WhatsApp and SMS text messages at the appropriate time points by the primary researcher (JYXC). The scales used were validated among the local population in previous studies [28,29,44]. Parenting self-efficacy was measured using the 4-point 10-item Parenting Efficacy Scale [45], while stress was measured by the 5-point 10-item Perceived Stress Scale [46]. Depression was measured by the 4-point 10-item Edinburgh Postnatal Depression Scale [47,48]. Moreover, the 4-point 40-item State-Trait Anxiety Inventory [49] and the 5-point 8-item Perceived Social Support for Parenting scale [45] were used to measure anxiety and social support, respectively. Finally, parent-child bonding was assessed by the 4-point 8-item Parent-Infant Bonding Questionnaire [50], and parenting satisfaction was measured by the 9-point 11-item evaluation subscale of the What Being the Parent of a Baby is Like scale [51]. All these scales have been validated for web-based administration in previous studies conducted among adults in Singapore [24,52].

### Use Metrics

PDA tracked each participant’s use and stored the information in a secure web-based administration portal that could only be accessed by researchers and technical experts. At the end of the intervention period, the use information was downloaded from the website, and 9 use metrics were extracted for analysis (Table 2). All metrics described the total number of various activities completed by participants during the study.

**Table 2:** Parentbot–a Digital Healthcare Assistant use metrics.

Use metric	Description
Number of educational materials	Total number of educational materials (text, video, or audio file) viewed by participants during the study, including repeated viewing of the same file
Number of chatbot questions	Total number of questions directed to the chatbot by participants during the study
Number of mindfulness videos	Total number of mindfulness-based meditation videos viewed by the participant during the study, including repeated viewing of the same file
Number of gratitude exercises	Total number of gratitude journal exercises completed by participants during the study
Number of reflection exercises	Total number of reflection journal exercises completed by participants during the study
Number of posters made	Total number of digital posters made by participants during the study
Number of poster likes given	Total number of likes given by participants to digital posters
Number of poster likes received	Total number of likes received by participants on their digital posters
Number of forum posts	Total number of forum posts made by participant

### Survey Response Timing

During the study, the primary researcher in charge of data collection (JYXC) recorded the date on which each participant received and completed each survey. At the end of the study, the number of days taken by each participant to complete the 3 surveys (baseline, 1 month post partum, and 3 months post partum) was calculated. When participants completed their survey on the same day that they received the survey link, their survey response timing was recorded as 0 days.

### Data Analysis

The SPSS software (version 29.0; IBM Corp) was used to perform all statistical analyses. The 2-sided statistical significance was set at *P*=.05. Categorical data were described using frequencies and percentages, while continuous data were described using means and SDs. The linear mixed model with a couple identification number as a random factor was used to examine the effect of (1) participants’ sociodemographic characteristics (ethnicity, gender, income, antenatal course attendance, education level, religion, age, and number of children) on use metrics, (2) use metrics on parenting outcomes (at 1 month and 3 months post partum), and (3) participants’ characteristics (grouping, ethnicity, gender, income, antenatal course attendance, education level, religion, age, and number of children) on survey response timing (at baseline, 1 month, and 3 months post partum).

To analyze the effect of participants’ characteristics on use metrics, univariate models were first used to examine the effect of each sociodemographic factor on each use metric. Next, multivariate models were used to examine the effect of all sociodemographic factors on each use metric. For the analysis of use metrics on parenting outcomes, two univariate models were used to examine the effect of each use metric on each parenting outcome: (1) adjusted for parenting outcome baseline values only and (2) adjusted for parenting outcome baseline values and covariates (ethnicity, gender, income, antenatal course attendance, education level, religion, age, and number of children). Next, multivariate models adjusted for both baseline values and covariates were used to examine the effect of all use metrics on each parenting outcome. To examine the effect of participants’ characteristics on survey response timing, univariate models were used to examine the effect of each variable on the survey response timing for each time point. Subsequently, multivariate models were used to examine the effect of all variables on each survey response timing. If no significant factor was identified by any multivariate model, an additional model consisting of only variables that had *P*<.10 in their respective univariate models would be built. The results from the univariate and multivariate models were compared to ascertain the presence of multicollinearity. Because no multicollinearity was detected for all analyses, this study only focused on the results obtained from the multivariate models. Moreover, to examine the linear relationships between use metrics and parenting outcomes at 1 and 3 months post partum, the Pearson correlation was calculated. All analyses were conducted using complete case data, but analyses related to the relationship between use metrics and parenting outcomes were also conducted using multiple imputed data based on the Markov chain Monte Carlo method (5 imputations) to assess the robustness of the models in the presence of missing data.

### Ethical Considerations

Ethics approval for the main RCT, which encompassed this secondary analysis, was obtained from the institution named National Healthcare Group Domain Specific Review Board, which is the institutional review board of the participating hospital (NHG2021/00227). Participants were informed about the study before giving their voluntary written consent. They were aware that they could withdraw from the study without any consequences. The participants’ anonymity was ensured by assigning each of them a unique identification number. The collected data were stored in password-protected laptops to maintain confidentiality. Each couple in the intervention group received SGD $30 (US $22.29) at baseline, SGD $30 (US $22.29) at 1 month post partum, and SGD $40 (US $29.71) at 3 months post partum if both parents completed the surveys at the respective time points. Each couple in the control group received SGD $15 (US $11.14) at baseline, SGD $15 (US $11.14) at 1 month post partum, and SGD $20 (US $14.85) at 3 months post partum if both parents completed the surveys at the respective time points. The money was sent to 1 parent from each couple according to the couple’s preference.

## Results

### Participants’ Characteristics

This study recruited 118 couples (236 participants: n=118, 50% mothers and n=118, 50% fathers), but 1.8% (2/114) of participants in the intervention group and 0.8% (1/117) of participants in the control group from different couples did not complete the baseline survey. The mean age of fathers in the intervention and control groups was 33.2 (SD 3.9) years and 34.1 (SD 4.4) years, respectively. The mean age of mothers in the intervention and control groups was 31.3 (SD 3.4) years and 31.8 (SD 3.9) years, respectively. A couple from the intervention group experienced a stillbirth and withdrew from the study. As a result, they were excluded from this study’s analysis, leaving 117 couples (234 participants) from both intervention (n=116, 49.6% participants) and control (n=118, 50.4% participants) groups. Most of the parents were Chinese (intervention: 46/114, 40.4% and control: 59/117, 50.4%), followed by Malay (intervention: 38/114, 33.3% and control: 37/117, 31.6%). More than half of the parents from the intervention (72/114, 63.2%) and control (78/117, 66.7%) groups had at least a Bachelor’s degree. More details on participants’ characteristics are presented in Multimedia Appendix 1.

**Figure 3:**
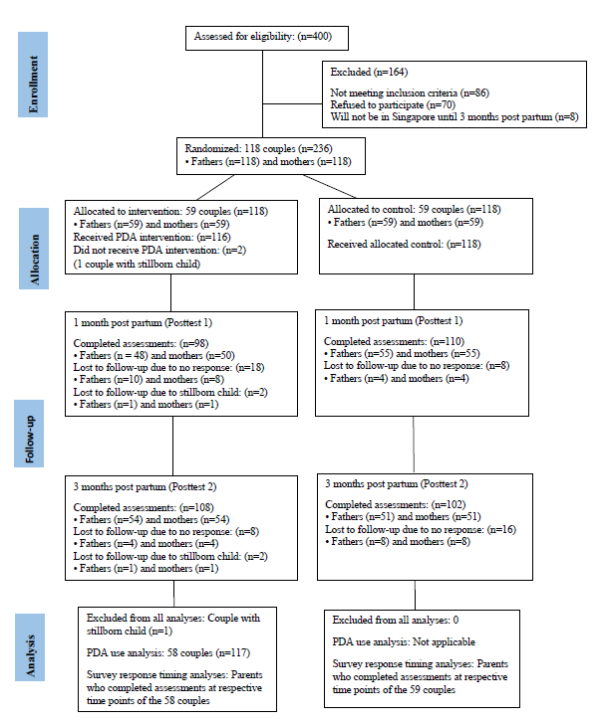
Parentbot–a Digital Healthcare Assistant (PDA) participants’ flow diagram.

Recruitment, follow-up data collection, and analysis from the participants in this study are shown using the CONSORT (Consolidated Standards of Reporting Trials) flow diagram in Figure 3. For PDA use analysis, data were analyzed from the 58 couples (116/118, 98.3% participants) in the intervention group, excluding the couple that withdrew.

For the survey response timing analysis, parents from both the intervention and control groups who completed their surveys at each time point (of the total 117 couples) were included: baseline—231 parents (n=116, 50.2% mothers and n=115, 49.8% fathers), 1 month post partum—208 parents (n=105, 50.5% mothers and n=103, 49.5% fathers), and 3 months post partum—210 parents (n=105, 50% mothers and n=105, 50% fathers).

### Use Metrics

PDA was available to the intervention group from >24 gestational weeks to 1 month post partum. Mothers (mean 8.9, SD 8.5) viewed more educational materials in PDA compared to fathers (mean 4.8, SD 5.7). Mothers (mean 14.4, SD 24.4) also directed more questions to the chatbot than fathers (mean 6.3, SD 9.9). On average, mothers and fathers completed similar numbers of gratitude, reflection, and mindfulness exercises; created a similar number of digital posters; received and gave a similar number of poster likes; and posted a similar number of posts onto the forum. Details on the use of metrics are presented in Multimedia Appendix 1.

### Effect of Participants’ Characteristics on PDA Use

**Figure 4:**
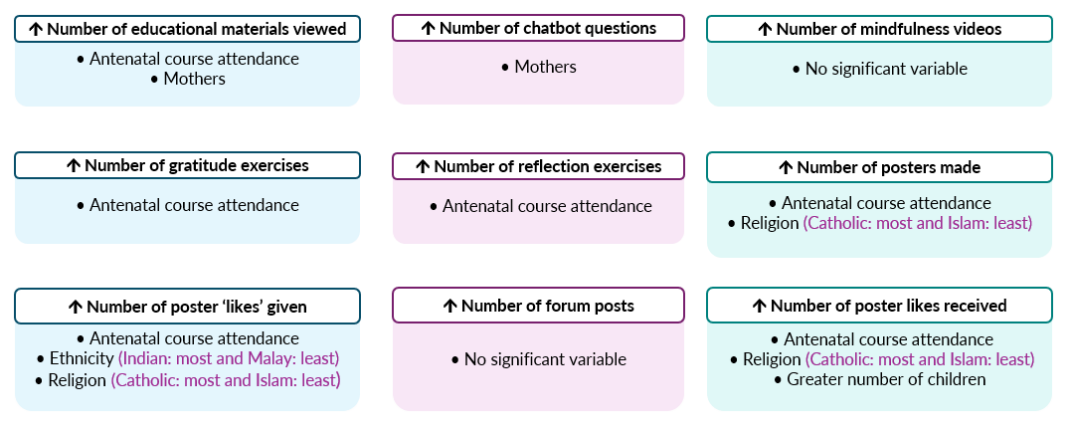
Visual summary of significant sociodemographic variables affecting Parentbot–a Digital Healthcare Assistant use. ↑: increase.

Results from the multivariate models showed that parents who attended antenatal courses viewed statistically significantly more educational materials (mean difference [MD] 4.46, 95% CI 0.51-8.41; *P*=.03), completed statistically significantly more gratitude (MD 1.33, 95% CI 0.17-2.48; *P*=.03) and reflection (MD 1.27, 95% CI 0.19-2.35; *P*=.02) journal exercises, made statistically significantly more posters (MD 2.25, 95% CI 0.89-3.61; *P*=.002), as well as gave (MD 4.35, 95% CI 1.01-7.68; *P*=.01) and received (MD 8.22, 95% CI 3.40-13.04; *P*=.001) statistically significantly more poster likes than those who did not attend any antenatal courses. Mothers reported viewing statistically significantly more educational materials (MD 6.20, 95% CI 2.83-9.57; *P*<.001) and asking the chatbot statistically significantly more questions (MD 9.77, 95% CI 1.08-18.46; *P*=.03) than fathers. Religion was identified as a statistically significant factor in the number of posters made (*F*_76_=2.26; *P*=.04) as well as the number of poster likes given (*F*_76_=3.41; *P*=.003) and received (*F*_76_=2.30, *P*=.04). Catholic parents completed the most poster activities (poster making and giving and receiving poster likes), while Muslim parents completed the least poster activities. Ethnicity was identified as a statistically significant factor in the number of poster likes given (*F*_76_=7.02, *P*<.001). Indian parents gave the greatest number of poster likes, while Malay parents gave the least number of poster likes. Moreover, parents with more children received statistically significantly more poster likes (β=4.03, 95% CI 0.21-7.85; *P*=.04). No statistically significant factor was identified for the number of mindfulness videos viewed and the number of forum posts. A visual summary of these results is presented in Figure 4, while more details are reported in Table S1 in Multimedia Appendix 2.

### Effect of PDA Use on Parenting Outcomes

#### One Month Post Partum: Complete Case Analysis

The results from the multivariate models are mentioned subsequently. Viewing more educational materials was only statistically significantly associated with lower anxiety (β=–0.48, 95% CI –0.94 to –0.009; *P*=.046), higher social support (β=0.31, 95% CI 0.08-0.54, *P*=.01), better parent-child bonding (β=–0.10, 95% CI –0.19 to –0.01; *P*=.03), and increased parenting satisfaction (β=0.57, 95% CI 0.07-1.07; *P*=.03). Directing more questions to the chatbot was only statistically significantly associated with higher depression (β=0.14, 95% CI 0.04-0.24; *P*=.007) and lower social support (β=–0.20, 95% CI –0.34 to –0.07; *P*=.004). The making of more posters was only statistically significantly associated with lower social support (β=–2.26, 95% CI –4.29 to –0.24; *P*=.03). Conversely, the viewing of mindfulness-based meditation videos, completing gratitude and reflection journal exercises, and giving and receiving poster likes and forum posts were not statistically significantly associated with any parenting outcome (Table S2 in Multimedia Appendix 2).

Parent-child bonding had statistically significant weak positive correlations with the number of chatbot questions (*r*=0.27; *P*=.007), number of gratitude exercises (*r*=0.23; *P*=.03), number of reflection exercises (*r*=0.20; *P*=.045), and number of poster likes given (*r*=0.24; *P*=.02). The number of forum posts had a statistically significant positive moderate correlation with parent-child bonding (*r*=0.32; *P*=.001) and a negative weak correlation with parenting satisfaction (*r*=–0.29, *P*=.003). The remaining correlations between the use metrics and parenting outcomes were weak and statistically nonsignificant (*r*=–0.18 to 0.19; *P*≥.05; Table S3 in Multimedia Appendix 2).

#### One Month Post Partum: Multiple Imputed Data

Similarly, results from the multiple imputed data identified no use metric significantly associated with parenting self-efficacy and stress. The number of educational materials viewed had similar significant relationships with anxiety and social support, while the number of questions directed to the chatbot had similar significant relationships with depression and social support. The number of posters made was also similarly significantly related to social support. However, no use metric was significantly associated with parent-child bonding and parenting satisfaction.

Similar weak and nonsignificant correlations were reported from the analysis of multiple imputed data between most use metrics and parenting outcomes. Parent-child bonding had similar significant weak positive correlations with the number of chatbot questions, the number of gratitude and reflection exercises, and the number of poster likes given. The number of forum posts had a similar positive moderate correlation with parent-child bonding and a negative weak correlation with parenting satisfaction.

#### Three Months Post Partum: Complete Case Analysis

Results from the multivariate models showed that none of the use metrics were statistically significantly associated with parenting self-efficacy, stress, anxiety, depression, and parent-child bonding. While no significant relationship was reported for any use metric in the main multivariate models for social support and parenting satisfaction, a few statistically significant relationships were reported in the additional models built with univariate factors of *P*<.10 for these 2 parenting outcomes. The number of poster likes given reported statistically significant negative relationships with parents’ social support (β=–0.23, 95% CI –0.45 to –0.01; *P*=.04) and parenting satisfaction (β=–0.50, 95% CI –0.84 to –0.16; *P*=.005). Moreover, the number of educational materials viewed had a statistically significant positive relationship with parenting satisfaction (β=0.31, 95% CI 0.01-0.61; *P*=.04; Table S4 in Multimedia Appendix 2).

The number of poster likes given had a statistically significant weak positive correlation (*r*=0.20; *P*=.04) with stress, while the number of forum posts had a statistically significant weak negative correlation (*r*=–0.21; *P*=.03) with anxiety. The remaining correlations between the use metrics and parenting outcomes were weak and statistically nonsignificant (*r*=–0.17 to 0.14; *P≥*.05; Table S5 in Multimedia Appendix 2).

#### Three Months Post Partum: Multiple Imputed Data

Results from the multiple imputed data similarly did not identify any significant relationship between the use metrics and parenting self-efficacy, stress, depression, and parent-child bonding. However, the number of forum posts had a significant negative relationship with anxiety, and no use metric had significant relationships with social support and parenting satisfaction (even in the additional models consisting of univariate factors of *P*<.10).

Results from the multiple imputed data showed similar weak and nonsignificant correlations between most use metrics and parenting outcomes. A similar significant weak positive correlation between the number of poster likes given and stress was reported. Moreover, the number of forum posts had a similar significant weak negative correlation with anxiety.

**Figure 5:**
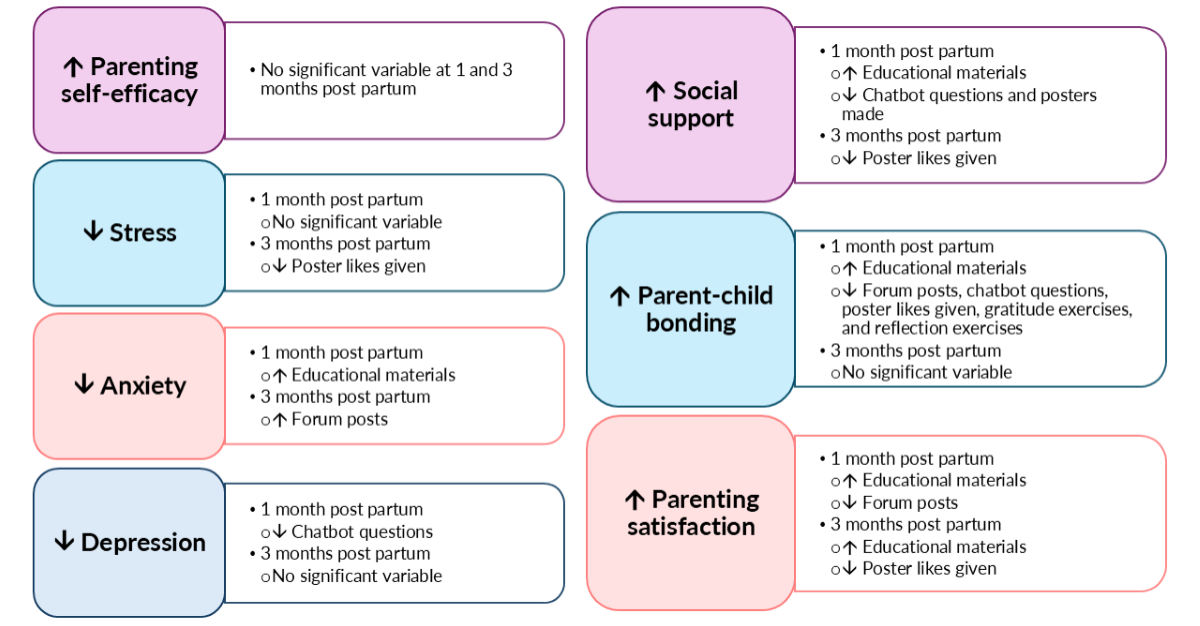
Visual summary of significant Parentbot–a Digital Healthcare Assistant use affecting parenting outcomes. ↑: increase; ↓: decrease.

A visual summary of all statistically significant results obtained from the complete case analysis at 1 month and 3 months post partum from the linear mixed model and correlation analyses is presented in Figure 5.

### Effect of Participants’ Characteristics on Survey Response Timing

**Figure 6:**
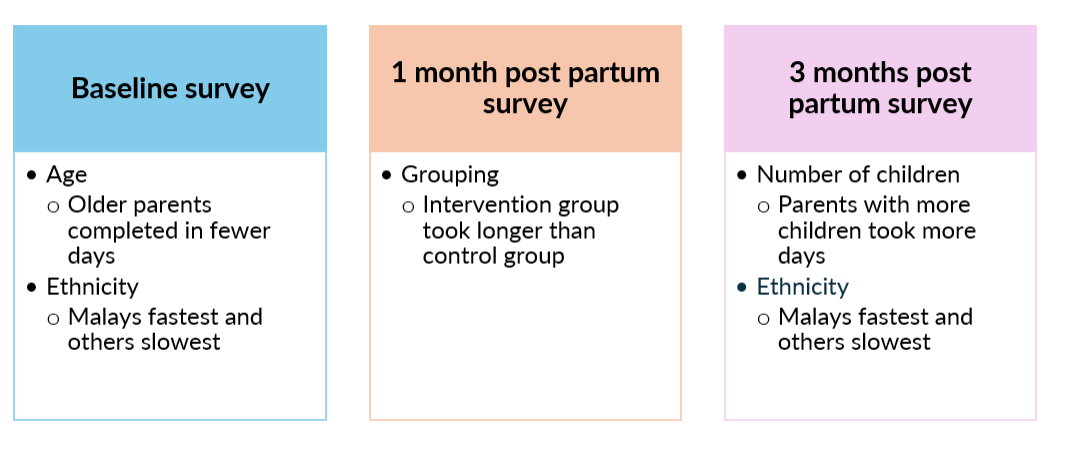
Visual summary of significant sociodemographic variables affecting survey response timing.

On average, participants took 1.22 (SD 4.74) days to complete the baseline survey, 6.58 (SD 8.86) days to complete the survey at 1 month post partum, and 5.05 (SD 8.16) days to complete the survey at 3 months post partum (Table S6 in Multimedia Appendix 2). Results from the multivariate models are mentioned subsequently. The survey response timing at baseline was statistically significantly affected by participants’ age (β=–0.15, 95% CI –0.28 to –0.021; *P*=.03) and ethnicity (*F*_185_=6.82; *P*<.001). Parents from the “others” ethnic group took the longest to complete their surveys, followed by Indian, Chinese, and Malay parents. The survey response timing at 1 month post partum was only statistically significantly affected by parents’ grouping; the intervention group took significantly longer than the control group to complete the survey (MD 3.51, 95% CI 1.03-5.99; *P*=.006). Finally, the survey response timing at 3 months post partum was statistically significantly affected by participants’ number of children (β=1.94, 95% CI 0.55-3.33; *P*=.007) and ethnicity (*F*_175.95_=5.06; *P*=.002). Malay parents were the fastest at completing their surveys, followed by Chinese and Indian parents and parents from the ‘others’ ethnic group. A visual summary of the statistically significant predictors of survey response timing is shown in Figure 6, while more details are reported in Table S7 in Multimedia Appendix 2.

## Discussion

### Principal Findings

This study examined the mobile app–based PDA intervention use, its relationship with parents’ sociodemographic data and parenting outcomes, and survey response timing by conducting a secondary analysis of an RCT. Results showed that parents’ antenatal course attendance, gender, religion, ethnicity, and number of children were significantly associated with their PDA use, thereby confirming the study’s hypothesis about significant relationships between participants’ characteristics and PDA use. Moreover, parents’ PDA use had some significant relationships with their parenting outcomes. Only the viewing of educational materials was associated with improvements in parents’ anxiety, parent-child bonding, social support, and parenting satisfaction. Conversely, other PDA activities, including communication with the chatbot, poster activities (making posters and giving and receiving poster likes), gratitude and reflection journal exercises, and forum posts were associated with poorer parenting outcomes, such as higher depression, lower social support, decreased parenting satisfaction, and poorer parent-child bonding. Mindfulness-based meditation was not associated with any parenting outcome. Because only the viewing of educational materials was associated with improved parenting outcomes while the remaining PDA activities were linked to poorer parenting outcomes or had no links to any parenting outcome, this study’s results only partially confirm the study’s hypothesis about the positive relationship between PDA use and improved parenting outcomes. Furthermore, the survey response timing was the longest at 1 month post partum and shortest at baseline. Parents’ age, ethnicity, grouping, and number of children were significantly related to the time taken to complete the trial’s surveys, thereby confirming the study’s hypothesis about significant relationships between participants’ characteristics and survey response timing.

The findings of this study reported that parents who attended antenatal courses viewed more educational materials, completed more gratitude and reflection journal exercises, and participated in more poster activities than parents who did not attend any courses. This could be due to first-time parents being more likely to attend antenatal courses as they seek more parenting-related knowledge [53], which could have led them to explore the PDA more often to fulfill their learning needs. Parents who attended antenatal courses could also possess more innate motivation to prepare themselves for the arrival of their new baby [54] or have more queries as they explore more knowledge and thus use the PDA more often to supplement their learning. Considering that parents can benefit from both the PDA and antenatal courses concurrently, health care providers could consider developing mobile app–based interventions, such as PDA, to supplement the current standard of perinatal care. This study also found that parents with more children received more poster likes as a sign of appreciation from other parents who valued their motivational messages. This is in line with previous local studies that reported parents benefiting from receiving peer support delivered by other experienced mothers serving as peer volunteers [55,56]. Because this study showed that both parents could benefit from receiving peer support, researchers could engage experienced mothers and fathers to serve as peer volunteers in future parenting interventions to promote more inclusivity for fathers in perinatal care.

Similar to previous studies that reported links between one’s religious and cultural beliefs and one’s perinatal and parenting practices [57,58], this study identified religion and ethnicity as significant factors of parents’ PDA use. Especially in multireligious and multiracial communities, such as Singapore, future researchers could collaborate with local religious and cultural leaders to develop culturally and spiritually appropriate resources for parents [57]. This study reported that mothers viewed more educational materials and used the chatbot function more often than fathers. These findings are similar to previous local and international studies where mothers were found to be more engaged in learning about pregnancy care, childbirth processes, and baby care skills than fathers [59,60]. However, as greater paternal involvement during the perinatal period has been linked to improved maternal and child health outcomes [61], future parenting interventions could also develop differentiated educational resources tailored to both parents’ perspectives and roles [62]. For instance, an educational resource on pregnancy nutrition for mothers could also be presented as “helping your partner eat right during pregnancy” for fathers. In addition, health care providers could receive special training on how to effectively engage fathers in maternal and childcare [63,64].

Similar to previous research [65,66], this study’s results showed that the viewing of educational materials was associated with positive parenting outcomes, including lowered anxiety, stronger parent-child bonds, and greater social support and parenting satisfaction. As education equips parents with valuable knowledge on how to take care of themselves and their newborns during pregnancy and after childbirth, the provision of such resources should be encouraged in future parenting interventions [12]. However, this finding contradicts the hypothesized positive association between the viewing of educational materials and parenting self-efficacy, especially since the self-efficacy theory by Bandura [33] posited that parents’ access to educational resources would encourage them to practice parenting skills and improve their parenting self-efficacy. This could be due to parents experiencing a lack of engagement with the educational materials; parents who used the PDA expressed their desire for games, quizzes, and personalized care tips provided at significant milestones during the perinatal period to be incorporated into the app [67]. As web-based games and the customization of mobile app–based interventions’ content could improve parents’ engagement in the intervention and facilitate their learning [68,69], future interventions could incorporate these elements. With the ability to provide users with near-authentic experiences, virtual reality could also be explored by future researchers to provide parents with a more immersive learning experience of childbirth and infant care [70,71].

The results of this study found that chatbot interaction, poster activities, gratitude and reflection journal exercises, and forum posts were generally associated with poor parenting outcomes. No links between the completion of mindfulness-based meditations and any parenting outcome were reported. These findings do not support the hypothesized positive association between PDA activities and improved parenting outcomes. This could be explained by parents’ perceived limitations of the PDA [67] and the low mean numbers (range 0.1-1.6) reported in this study for poster making, gratitude and reflection journal exercises, forum posts, and mindfulness-based meditations. Parents mentioned having problems communicating with the chatbot and too little activity on the discussion forum [67]. Some also commented on losing interest in some activities, such as poster making and gratitude and reflection journal exercises, over time [67]. The chatbot’s lack of artificial intelligence could have caused it to provide parents with irrelevant answers to their queries and did not facilitate their learning of perinatal care knowledge, a common problem noted by a previous review on chatbots [72]. In addition, the text-based chatbot in the PDA could be limited in its ability to simulate authentic real-life conversations with people and make parents feel less supported [73]. Thus, future chatbots could be programmed to have artificial intelligence and embodied conversational agents (animated avatars with speech, text, and nonverbal features) instead of allowing parents to have more realistic and satisfying conversations with them [74,75]. The lack of activity in the support forum could also have prevented parents from receiving valuable social support; hence, future researchers could introduce weekly topics and initiate regular posts to increase participants’ engagement in the forum [76]. To address parents’ lack of sustained interest in poster making, gratitude and reflection journal exercises, and mindfulness-based meditations, future interventions could explore the use of other more engaging and interactive features, such as games, and tailored push notifications to promote the psychosocial well-being of parents instead [77,78]. In particular, having more engaging and interactive games could help to enhance parents’ well-being and empower parents to play a more active and engaged role in caring for their infants [79]. Moreover, because open-world games tend to immerse players in extensive and interactive environments to shift their attention away from stressors in real life through a process known as cognitive escapism, parents could appreciate having such games added to future variants of the PDA intervention to help them relax and improve their psychological well-being [80].

This study found that parents took the longest time to complete the survey at 1 month post partum, possibly because they were busy managing various challenges while recovering from the labor and childbirth experience and adjusting to life with their new babies [56]. Because the delayed completion of research surveys could affect the quality of data collected [25,81], future studies on parents during the perinatal period could find creative ways on how data at such a crucial postpartum time point can still be collected. Interviewing local multiracial parents may help researchers gain some insights into the best ways to collect data from them at 1 month post partum, as this is also the period when they practice traditional confinement (doing the month) rituals. These confinement practices typically involve mothers resting at home for the first month post partum and following specific dietary requirements to consume nourishing meals believed to aid recovery after labor [82,83].

Similar to previous studies [84,85], the findings of this study also indicated that older parents provided more prompt survey responses compared to their younger counterparts. This could be due to older parents having more maturity to cope with challenges, such as raising newborns, and hence being more able to respond to surveys promptly [86,87]. Parents with more children were found to take longer to complete their surveys, possibly because they were busy caring for multiple children and attending to other responsibilities and had less spare time to complete surveys. Ethnicity was also revealed to affect parents’ survey response timing; Malay parents took the shortest time to complete their surveys, followed by the Chinese and Indian parents and parents from the ‘others’ ethnic group. As parents’ perinatal practices are influenced by their cultural beliefs [57,58], parents could be preoccupied with their traditional cultural practices related to maternal and child health and have less spare time to complete their surveys. Hence, researchers would need to be more aware of the various cultural practices adopted by the local multiracial parents and be patient when certain groups of parents take longer to complete their surveys.

Moreover, this study found that parents from the intervention group took a longer time to complete their surveys than the control group immediately after the intervention at 1 month post partum. This could be due to research study fatigue, as they were involved in the PDA intervention until 1 month post partum and had received regular weekly reminders from the researchers reminding them to use the app from pregnancy until 1 month post partum [88]. When the intervention ended, the parents might have missed out on reading the message from the researchers informing them to complete the web-based survey or be too tired to complete the survey promptly. In addition, the multiple studies being conducted on the same group of parents at the tertiary public hospital could have contributed to parents’ research fatigue [88]. Hence, researchers planning to recruit participants from the same setting need to plan their study timelines carefully so as not to burden the same pool of individuals participating in multiple research studies concurrently [88].

### Strengths and Limitations

By only including English-speaking heterosexual couples who had babies without serious medical complications from one public hospital, this study’s findings cannot be generalized to all parents in Singapore. Because all participants identified as heterosexual, the results of this study might not represent parents from the lesbian, gay, bisexual, and transgender community. The results obtained could be at risk for social desirability bias, as self-reported surveys were used to collect data on parenting outcomes. As minor differences between the results obtained from the complete case analysis and multiple imputed data were identified, this study’s findings could have inadequate robustness, and more studies are needed to validate the results of this study [89]. Moreover, as this study only measured PDA use by monitoring parents’ completion of activities, conclusions regarding the frequency and duration of parents’ PDA use could not be drawn. The measurement of PDA use could also have some inaccuracies, given that the tracker was not able to differentiate between parents’ attempts (eg, starting a video on baby bathing) and completion (eg, finishing viewing the entire video on baby bathing) of each activity. Despite these limitations, this was the first study to analyze parents’ use of a mobile app–based intervention and the time taken to respond to surveys during the trial. The results of this study could provide valuable insights for researchers to develop future mobile app–based parenting interventions that prioritize the efficiency of the most useful features (eg, educational resources) and improve other features (eg, chatbot, gratitude, and reflection journal exercises) to make them more attractive and interactive for local parents. The identified relationships between parents’ sociodemographic characteristics and PDA use as well as survey response timing could inform future researchers of certain groups of study participants who may require more motivation to participate in research studies.

### Implications for Future Research and Practice

This study showed that parents’ active perusal of educational resources via app-based interventions could improve their perinatal well-being. Hence, future parenting interventions should continue providing informational support to parents via such apps. However, the use of other PDA features, such as the chatbot, gratitude and reflection exercises, discussion forum, and mindfulness-based meditations, was not as successful in improving parents’ well-being. Thus, future interventions could explore more interactive and attractive features, such as games, simulations, virtual reality technology, and embodied conversational agents to provide psychosocial support to parents. Gamification features could especially be leveraged to enhance parents’ well-being, and the potential of using open-world games to improve parents’ emotional well-being could be further explored as well. To provide parents with more peer support, both experienced fathers and mothers could be recruited to serve as peer volunteers in future interventions. As fathers need more encouragement to participate in perinatal care, health care providers could receive specialized training on engaging fathers, and future interventions could have more resources tailored to fathers’ needs. Given Singapore’s multiethnic and multireligious society, local parents would benefit from having more culturally and spiritually appropriate resources. Furthermore, because PDA can enhance parents’ learning of perinatal knowledge even after they attend antenatal courses, health care professionals could consider using a similar mobile app–based intervention to supplement the current standard perinatal care.

To obtain a more comprehensive understanding of parents’ app use, future trials should monitor participants’ frequency and duration of use together with their completion of activities. More sophisticated tracking systems could also be used to differentiate between participants’ attempts at and completion of activities. For more timely survey responses, future researchers would need to explore more efficient methods of collecting data from parents, especially at 1 month post partum, where delays are more common. Researchers would also need to be mindful of the local community’s perinatal cultural practices that could hinder their participation in trials. Finally, to prevent participants from experiencing research study fatigue, careful collaboration among researchers who plan to recruit participants from the same setting would be required.

### Conclusions

Overall, this study found that the viewing of educational resources was associated with better parenting outcomes, while other PDA activities, such as poster making and forum posts, had limited effect on enhancing parents’ perinatal well-being. Some parents’ sociodemographic characteristics were found to be associated with their PDA use and survey response timing. Researchers could use the findings of this study to guide future parenting interventions by prioritizing effective components and improving less popular features. Future parenting interventions could be more interactive, engaging, culturally and spiritually appropriate, and inclusive of fathers. In addition, future trials of mobile app–based interventions for parents could analyze app use and survey response timing to corroborate the findings of this study.

## Appendix

Multimedia Appendix 1 Participants’ characteristics.

Multimedia Appendix 2 Tables on Parentbot–a Digital Healthcare Assistant use and survey response timing.

## Abbreviations

CONSORT: Consolidated Standards of Reporting Trials
MD: mean difference
PDA: Parentbot–a Digital Healthcare Assistant
RCT: randomized controlled trial
STROBE: Strengthening the Reporting of Observational Studies in Epidemiology

## Data Availability

All data generated or analyzed during this study are included in this published article and its supplementary information files.
