# Supplementary material for: A Mobile App–Based Intervention (Parentbot–a Digital Healthcare Assistant) for Parents: Secondary Analysis of a Randomized Controlled Trial
Source: J Med Internet Res. 2025 Apr 17;27:e64882. doi: 10.2196/64882 (PMC12046274; doi:10.2196/64882)
Supplement: Multimedia Appendix 1 [file jmir_v27i1e64882_app1.docx]

**Supplementary table of participants' characteristics**

Table of participants’ characteristics and Parentbot–a Digital healthcare Assistant (PDA) use metrics among parents

| Characteristics | | Intervention group | | Control group | |
| --- | --- | --- | --- | --- | --- |
|  | | Mothers (n=58) | Fathers (n=58) | Mothers (n=59) | Fathers (n=59) |
|  | | | | | |
| Age (y), mean (SD; range) | | 31.3 (3.4; 24-39) | 33.2 (3.9; 26-45) | 31.8 (3.9; 23-41) | 34.1 (4.4; 25-48) |
| **Ethnicity, n^a^ (%)** | | | | | |
|  | Chinese | 24 (41) | 22 (38) | 30 (51) | 29 (50) |
|  | Indian | 6 (10) | 8 (14) | 3 (5) | 4 (7) |
|  | Malay | 19 (33) | 19 (33) | 19 (32) | 18 (31) |
|  | Others | 8 (14) | 8 (14) | 7 (12) | 7 (12) |
| **Religion, n^a^ (%)** | | | | | |
|  | Buddhism | 7 (12) | 6 (10) | 9 (15) | 7 (12) |
|  | Taoism | 3 (5) | 4 (7) | 0 (0) | 2 (3) |
|  | Hinduism | 2 (3) | 4 (7) | 1 (2) | 2 (3) |
|  | Christianity | 9 (16) | 6 (10) | 9 (15) | 6 (10) |
|  | Catholicism | 5 (9) | 2 (3) | 3 (5) | 1 (2) |
|  | Islam | 25 (43) | 24 (41) | 24 (41) | 23 (39) |
|  | No religion | 4 (7) | 9 (16) | 12 (20) | 16 (27) |
|  | Others | 2 (3) | 2 (3) | 1 (2) | 1 (2) |
| **Marital status, n^a^ (%)** | | | | | |
|  | Married | 56 (97) | 56 (97) | 59 (100) | 58 (100) |
|  | Single | 1 (2) | 1 (2) | 0 (0) | 0 (0) |
| Length of marriage (y), mean (SD; range) | | 4.3 (3.0; 1-13) | 4.3 (3.0; 1-13) | 4.8 (3.0; 1-14) | 4.9 (3.0; 1-14) |
| **Education level, n^a^ (%)** | | | | | |
|  | Secondary school | 3 (5) | 3 (5) | 2 (3) | 8 (14) |
|  | ITE^b^, polytechnic, or junior college | 15 (26) | 21 (36) | 14 (24) | 15 (25) |
|  | University degree | 33 (57) | 22 (38) | 24 (41) | 20 (34) |
|  | Postgraduate degree | 6 (10) | 11 (19) | 19 (32) | 15 (25) |
| **Employment status, n^a^ (%)** | | | | | |
|  | Employed | 51 (88) | 55 (95) | 50 (85) | 57 (97) |
|  | Unemployed | 6 (10) | 2 (3) | 9 (15) | 1 (2) |
| **Monthly household income (SGD), n^a^ (%)** | | | | | |
|  | <1000 (<US $743) | 0 (0) | 1 (2) | 1 (2) | 1 (2) |
|  | 1000-3000  (US $743-$2,229) | 7 (12) | 5 (9) | 7 (12) | 6 (10) |
|  | 3000-5000  (US $2,229-$3,715) | 11 (19) | 14 (24) | 9 (15) | 13 (22) |
|  | 5000-7000  (US $3715-$5201) | 11 (19) | 10 (17) | 9 (15) | 4 (7) |
|  | 7000-10,000  (US $5201-$7,430) | 15 (26) | 13 (22) | 12 (20) | 12 (20) |
|  | >10,000 (>US $7430) | 13 (22) | 14 (24) | 21 (36) | 22 (37) |
| **Baby’s sex, n^a^ (%)** | | | | | |
|  | Male | 27 (52.9) | 27 (56.3) | 27 (49.1) | 29 (52.7) |
|  | Female | 24 (47.1) | 21 (43.8) | 28 (50.9) | 26 (47.3) |
| **Childbirth method, n^a^ (%)** | | | | | |
|  | Normal vaginal delivery | 23 (45.1) | 21 (43.8) | 35 (63 6) | 36 (65.5) |
|  | Assisted vaginal delivery using vacuum extraction | 4 (7.8) | 3 (6.3) | 2 (3.6) | 2 (3.6) |
|  | LSC^c^ (emergency) | 15 (29.4) | 14 (29.2) | 10 (18.2) | 9 (16.4) |
|  | LSC (elective) | 9 (17.6) | 10 (20.8) | 8 (14.5) | 8 (14.5) |
|  | Attended antenatal preparation courses | 14 (27.5) | 13 (27.1) | 11 (20) | 11 (20) |
| **Mothers’ confinement period (wk), n^a^ (%)** | | | | | |
|  | <2 | 3 (5.9) | N/A^c^ | 1 (1.8) | N/A |
|  | 2-4 | 19 (37.3) | N/A | 24 (43.6) | N/A |
|  | 4-6 | 18 (35.3) | N/A | 22 (40) | N/A |
|  | >6 | 6 (11.8) | N/A | 2 (3.6) | N/A |
|  | None | 5 (9.8) | N/A | 6 (10.9) | N/A |
| **Method of feeding baby, n^a^ (%)** | | | | | |
|  | Breast milk only (refers to both direct breastfeeding and expressed breast milk) | 22 (43.1) | 20 (41.7) | 16 (29.1) | 19 (34.5) |
|  | Formula feeds only | 5 (9.8) | 4 (8.3) | 3 (5.5) | 4 (7.3) |
|  | Mixture of breast milk and formula feeds | 24 (47.1) | 24 (50.0) | 36 (65.5) | 32 (58.2) |
| **Length of maternity or paternity leave, n^a^ (%)** | | | | | |
|  | 1 wk | 0 (0) | 1 (2.1) | 0 (0) | 7 (12.7) |
|  | 2 wk | 0 (0) | 30 (62.5) | 0 (0) | 29 (52.7) |
|  | 3-4 wk | 0 (0) | 4 (8.3) | 1 (1.8) | 10 (18.2) |
|  | 1-2 mo | 3 (5.9) | 4 (8.3) | 2 (3.6) | 2 (3.6) |
|  | 2-3 mo | 4 (7.8) | 0 (0) | 3 (5.5) | 1 (1.8) |
|  | 3-4 mo | 31 (60.8) | 0 (0) | 34 (61.8) | 0 (0) |
|  | >4 mo | 7 (13.7) | 0 (0) | 4 (7.3) | 1 (1.8) |
|  | Did not take leave | 1 (2.0) | 8 (16.7) | 1 (1.8) | 4 (7.3) |
|  | Not applicable (not currently working) | 5 (9.8) | 1 (2.1) | 10 (18.2) | 1 (1.8) |
| **Total number of children (including new baby), n^a^ (%)** | | | | | |
|  | 1 | 27 (52.9) | 24 (50.0) | 29 (52.7) | 30 (54.5) |
|  | 2 | 21 (41.2) | 21 (43.8) | 17 (30.9) | 16 (29.1) |
|  | ≥3 | 3 (5.9) | 3 (6.3) | 9 (16.4) | 9 (16.4) |
| **PDA use metrics, mean (SD; range)** | | | | | |
|  | Number of educational materials | 8.9 (8.5; 0-44) | 4.8 (5.7; 0-20) | N/A | N/A |
|  | Number of chatbot questions | 14.4 (24.4; 0-107) | 6.3 (9.9; 0-48) | N/A | N/A |
|  | Number of mindfulness videos | 1.5 (2.4; 0-12) | 1.5 (2.8; 0-11) | N/A | N/A |
|  | Number of gratitude exercises | 1.2 (2.4; 0-12) | 0.8 (1.9; 0-11) | N/A | N/A |
|  | Number of reflection exercises | 1.1 (2.1; 0-10) | 0.8 (1.8; 0-11) | N/A | N/A |
|  | Number of posters made | 1.6 (3.0; 0-14) | 1.0 (2.0; 0-12) | N/A | N/A |
|  | Number of poster “likes” given | 2.9 (8.1; 0-49) | 2.4 (5.3; 0-27) | N/A | N/A |
|  | Number of poster “likes” received | 5.8 (10.8; 0-47) | 3.5 (7.4; 0-38) | N/A | N/A |
|  | Number of forum posts | 0.3 (1.3; 0-9) | 0.1 (0.4; 0-3) | N/A | N/A |

^a^Total number of mothers and fathers vary across different characteristics due to missing survey responses and incomplete survey responses

^b^ITE: Institute of Technical Education

^c^LSC: lower segment cesarean

^d^Not available
