# Supplementary material for: A Mobile App–Based Intervention (Parentbot–a Digital Healthcare Assistant) for Parents: Secondary Analysis of a Randomized Controlled Trial
Source: J Med Internet Res. 2025 Apr 17;27:e64882. doi: 10.2196/64882 (PMC12046274; doi:10.2196/64882)
Supplement: Multimedia Appendix 2 [file jmir_v27i1e64882_app2.docx]

**Supplementary tables** **on PDA use and survey response timing**

**Table S1.** Significant sociodemographic variables affecting PDA use among intervention group parents in multivariate models

| **Variable** | **Mean (Standard deviation)** | **MD (95%CI) or β (95%CI)** | **F-value** | **P-value** |
| --- | --- | --- | --- | --- |
| **Number of educational materials viewed** | | | | |
| Antenatal course attendance |  |  | 5.07 | 0.027 |
| Attended course | 10.74 (10.51) | 4.46 (0.51 to 8.41) |  |  |
| Did not attend course* | 6.18 (6.00) |  |  |  |
| Parent |  |  | 13.45 | <0.001 |
| Mother | 8.93 (8.49) | 6.20 (2.83 to 9.57) |  |  |
| Father* | 4.75 (5.71) |  |  |  |
| **Number of questions directed to chatbot** | | | | |
| Parent |  |  | 5.01 | 0.028 |
| Mother | 14.35 (24.38) | 9.77 (1.08 to 18.46) |  |  |
| Father* | 6.32 (9.87) |  |  |  |
| **Number of mindfulness videos viewed** | | | | |
| No significant variable identified in multivariate model |  |  |  |  |
| No significant variable identified in model consisting of univariate factors with p-value < 0.1 |  |  |  |  |
| **Number of gratitude exercises completed** | | | | |
| Antenatal course attendance |  |  | 5.25 | 0.025 |
| Attended course | 1.74 (2.64) | 1.33 (0.17 to 2.48) |  |  |
| Did not attend course* | 0.92 (2.11) |  |  |  |
| **Number of reflection exercises completed** | | | | |
| Antenatal course attendance |  |  | 5.48 | 0.022 |
| Attended course | 1.63 (2.62) | 1.27 (0.19 to 2.35) |  |  |
| Did not attend course* | 0.83 (1.85) |  |  |  |
| **Number of posters made** | | | | |
| Antenatal course attendance |  |  | 10.81 | 0.002 |
| Attended course | 2.52 (3.56) | 2.25 (0.89 to 3.61) |  |  |
| Did not attend course* | 1.08 (2.25) |  |  |  |
| Religion |  |  | 2.26 | 0.038 |
| Buddhism | 1.58 (2.68) | 0.19 (-2.14 to 2.52) |  | 0.871 |
| Taoism | 1.20 (2.68) | -0.17 (-3.21 to 2.86) |  | 0.910 |
| Hinduism | 3.33 (5.32) | -2.94 (-7.36 to 1.48) |  | 0.190 |
| Christianity | 2.07 (3.71) | 0.95 (-1.01 to 2.90) |  | 0.339 |
| Catholicism | 3.43 (4.69) | 1.09 (-1.60 to 3.77) |  | 0.424 |
| Islam | 0.68 (1.04) | -5.54 (-9.42 to -1.65) |  | 0.006 |
| Others | 1.17 (1.34) | 0.13 (-3.18 to 3.44) |  | 0.939 |
| No religion* | 1.50 (2.38) |  |  |  |
| **Number of poster ‘likes’ given** | | | | |
| Antenatal course attendance |  |  | 6.73 | 0.011 |
| Attended course | 5.56 (8.97) | 4.35 (1.01 to 7.68) |  |  |
| Did not attend course* | 1.99 (6.28) |  |  |  |
| Ethnicity |  |  | 7.02 | <0.001 |
| Chinese | 2.20 (4.61) | -6.93 (-12.08 to -1.77) |  | 0.009 |
| Malay | 0.68 (1.47) | 9.52 (1.09 to 17.96) |  | 0.027 |
| Indian | 6.71 (13.79) | 15.69 (7.11 to 24.28) |  | <0.001 |
| Others* | 4.94 (9.23) |  |  |  |
| Religion |  |  | 3.41 | 0.003 |
| Buddhism | 4.25 (8.01) | 2.14 (-3.57 to 7.85) |  | 0.458 |
| Taoism | 1.60 (3.58) | -2.12 (-9.56 to 5.32) |  | 0.572 |
| Hinduism | 5.50 (9.46) | -18.20 (-29.04 to -7.35) |  | 0.001 |
| Christianity | 3.80 (8.87) | 1.45 (-3.35 to 6.25) |  | 0.548 |
| Catholicism | 9.14 (17.93) | 0.37 (-6.22 to 6.96) |  | 0.912 |
| Islam | 1.00 (1.90) | -18.42 (-27.94 to -8.90) |  | <0.001 |
| Others | 2.92 (4.80) | -4.09 (-12.21 to 4.03) |  | 0.319 |
| No religion* | 1.75 (2.87) |  |  |  |
| **Number of poster ‘likes’ received** | | | | |
| Antenatal course attendance |  |  | 11.53 | 0.001 |
| Attended course | 9.00 (11.53) | 8.22 (3.40 to 13.04) |  |  |
| Did not attend course* | 3.92 (8.70) |  |  |  |
| Religion |  |  | 2.30 | 0.035 |
| Buddhism | 5.58 (11.02) | 0.25 (-8.00 to 8.50) |  | 0.952 |
| Taoism | 5.60 (12.52) | 1.25 (-9.49 to 12.00) |  | 0.817 |
| Hinduism | 11.00 (17.33) | -10.25 (-25.92 to 5.41) |  | 0.196 |
| Christianity | 7.60 (11.33) | 3.39 (-3.55 to 10.32) |  | 0.334 |
| Catholicism | 14.29 (17.90) | 6.81 (-2.71 to 16.32) |  | 0.158 |
| Islam | 2.16 (3.71) | -17.78 (-31.54 to -4.02) |  | 0.012 |
| Others | 4.25 (4.81) | 0.63 (-11.10 to 12.36) |  | 0.915 |
| No religion* | 4.25 (7.23) |  |  |  |
| Number of children |  | 4.03 (0.21 to 7.85) | 4.41 | 0.039 |
| **Number of posts on forum** | | | | |
| No significant variable identified in multivariate model |  |  |  |  |
| No additional model built as all univariate models had p-value >0.1 |  |  |  |  |
| ***Note:***  *Reference group; CI: Confidence interval; MD: Mean difference; β: Unstandardized estimate of fixed effect  Multivariate models are adjusted for the following variables: ethnicity, gender, income, antenatal course attendance, education level, religion, age, and number of children. All MD (95%CI)s are calculated based on the estimated marginal means. | | | | |

**Table S2.** Regression estimates of PDA use on parenting outcomes at one month postpartum among intervention group parents in multivariate models

| **Variable** | **β (95%CI)** | **Standard Error (SE)** | **t-value** | **p-value** |
| --- | --- | --- | --- | --- |
| **Outcome 1: Parenting self-efficacy** | | | | |
| Number of educational materials | 0.01 (-0.17 to 0.19) | 0.09 | 0.10 | 0.922 |
| Number of chatbot questions | -0.08 (-0.19 to 0.03) | 0.05 | -1.52 | 0.133 |
| Number of mindfulness videos | -0.29 (-0.87 to 0.30) | 0.29 | -0.98 | 0.331 |
| Number of gratitude exercises | 0.09 (-4.07 to 4.24) | 2.08 | 0.04 | 0.967 |
| Number of reflection exercises | 0.67 (-3.25 to 4.58) | 1.96 | 0.34 | 0.735 |
| Number of posters made | -0.48 (-2.06 to 1.11) | 0.80 | -0.60 | 0.553 |
| Number of poster ‘likes’ given | 0.01 (-0.34 to 0.35) | 0.17 | 0.05 | 0.962 |
| Number of poster ‘likes’ received | 0.15 (-0.26 to 0.56) | 0.21 | 0.72 | 0.472 |
| Number of forum posts | 0.02 (-1.06 to 1.09) | 0.54 | 0.03 | 0.974 |
| **Outcome 2: Stress** | | | | |
| Number of educational materials | -0.06 (-0.31 to 0.20) | 0.13 | -0.44 | 0.660 |
| Number of chatbot questions | 0.05 (-0.10 to 0.20) | 0.07 | 0.65 | 0.517 |
| Number of mindfulness videos | -0.12 (-0.90 to 0.66) | 0.39 | -0.30 | 0.766 |
| Number of gratitude exercises | 1.18 (-4.46 to 6.83) | 2.83 | 0.42 | 0.677 |
| Number of reflection exercises | -1.00 (-6.29 to 4.30) | 2.65 | -0.38 | 0.708 |
| Number of posters made | 1.22 (-0.92 to 3.35) | 1.07 | 1.14 | 0.260 |
| Number of poster ‘likes’ given | -0.25 (-0.71 to 0.20) | 0.23 | -1.11 | 0.270 |
| Number of poster ‘likes’ received | -0.24 (-0.80 to 0.32) | 0.28 | -0.85 | 0.398 |
| Number of forum posts | -0.83 (-2.27 to 0.62) | 0.72 | -1.14 | 0.257 |
| **Outcome 3: Anxiety** | | | | |
| Number of educational materials | -0.48 (-0.94 to -0.009) | 0.23 | -2.03 | 0.046* |
| Number of chatbot questions | 0.05 (-0.24 to 0.33) | 0.14 | 0.32 | 0.752 |
| Number of mindfulness videos | 0.34 (-1.16 to 1.84) | 0.75 | 0.45 | 0.651 |
| Number of gratitude exercises | 3.04 (-7.88 to 13.96) | 5.47 | 0.56 | 0.580 |
| Number of reflection exercises | -3.58 (-13.76 to 6.60) | 5.10 | -0.70 | 0.485 |
| Number of posters made | 0.34 (-3.74 to 4.41) | 2.04 | 0.16 | 0.870 |
| Number of poster ‘likes’ given | -0.53 (-1.40 to 0.35) | 0.44 | -1.21 | 0.231 |
| Number of poster ‘likes’ received | 0.36 (-0.71 to 1.43) | 0.54 | 0.67 | 0.506 |
| Number of forum posts | -1.69 (-4.52 to 1.14) | 1.42 | -1.19 | 0.237 |
| **Outcome 4: Depression** | | | | |
| Number of educational materials | -0.12 (-0.28 to 0.03) | 0.08 | -1.58 | 0.118 |
| Number of chatbot questions | 0.14 (0.04 to 0.24) | 0.05 | 2.80 | 0.007* |
| Number of mindfulness videos | 0.16 (-0.35 to 0.68) | 0.26 | 0.64 | 0.526 |
| Number of gratitude exercises | -1.80 (-5.57 to 1.96) | 1.89 | -0.96 | 0.342 |
| Number of reflection exercises | 1.29 (-2.25 to 4.82) | 1.77 | 0.73 | 0.470 |
| Number of posters made | -0.22 (-1.61 to 1.18) | 0.70 | -0.31 | 0.759 |
| Number of poster ‘likes’ given | 0.16 (-0.14 to 0.45) | 0.15 | 1.06 | 0.292 |
| Number of poster ‘likes’ received | -0.10 (-0.47 to 0.27) | 0.19 | -0.54 | 0.594 |
| Number of forum posts | 0.03 (-0.95 to 1.01) | 0.49 | 0.07 | 0.947 |
| **Outcome 5: Social support** | | | | |
| Number of educational materials | 0.31 (0.08 to 0.54) | 0.12 | 2.65 | 0.01* |
| Number of chatbot questions | -0.20 (-0.34 to -0.07) | 0.07 | -2.95 | 0.004* |
| Number of mindfulness videos | 0.35 (-0.39 to 1.09) | 0.37 | 0.95 | 0.345 |
| Number of gratitude exercises | 4.47 (-0.92 to 9.86) | 2.70 | 1.66 | 0.102 |
| Number of reflection exercises | -4.00 (-9.04 to 1.05) | 2.53 | -1.58 | 0.119 |
| Number of posters made | -2.26 (-4.29 to -0.24) | 1.01 | -2.23 | 0.029* |
| Number of poster ‘likes’ given | 0.24 (-0.19 to 0.67) | 0.22 | 1.12 | 0.266 |
| Number of poster ‘likes’ received | 0.48 (-0.06 to 1.02) | 0.27 | 1.79 | 0.077 |
| Number of forum posts | 0.18 (-1.19 to 1.55) | 0.68 | 0.26 | 0.792 |
| **Outcome 6: Parent-child bonding** | | | | |
| Number of educational materials | -0.10 (-0.19 to -0.01) | 0.05 | -2.27 | 0.027* |
| Number of chatbot questions | 0.04 (-0.01 to 0.10) | 0.03 | 1.60 | 0.115 |
| Number of mindfulness videos | -0.14 (-0.43 to 0.15) | 0.15 | -0.95 | 0.35 |
| Number of gratitude exercises | -1.49 (-3.59 to 0.61) | 1.05 | -1.42 | 0.161 |
| Number of reflection exercises | 1.49 (-0.48 to 3.46) | 0.99 | 1.51 | 0.136 |
| Number of posters made | 0.35 (-0.44 to 1.14) | 0.40 | 0.88 | 0.383 |
| Number of poster ‘likes’ given | 0.04 (-0.13 to 0.21) | 0.09 | 0.49 | 0.627 |
| Number of poster ‘likes’ received | -0.07 (-0.28 to 0.14) | 0.10 | -0.67 | 0.503 |
| Number of forum posts | 0.29 (-0.27 to 0.84) | 0.28 | 1.03 | 0.308 |
| **Outcome 7: Parenting satisfaction** | | | | |
| Number of educational materials | 0.57 (0.07 to 1.07) | 0.25 | 2.27 | 0.027* |
| Number of chatbot questions | -0.12 (-0.42 to 0.18) | 0.15 | -0.83 | 0.412 |
| Number of mindfulness videos | 0.29 (-1.31 to 1.88) | 0.80 | 0.36 | 0.722 |
| Number of gratitude exercises | -6.01 (-17.58 to 5.56) | 5.79 | -1.04 | 0.304 |
| Number of reflection exercises | 5.44 (-5.41 to 16.30) | 5.43 | 1.00 | 0.320 |
| Number of posters made | -3.62 (-8.07 to 0.82) | 2.23 | -1.63 | 0.109 |
| Number of poster ‘likes’ given | 0.39 (-0.55 to 1.33) | 0.47 | 0.82 | 0.414 |
| Number of poster ‘likes’ received | 0.99 (-0.16 to 2.13) | 0.58 | 1.71 | 0.091 |
| Number of forum posts | -1.26 (-4.34 to 1.83) | 1.54 | -0.81 | 0.419 |
| ***Note:***  *significant result (p-value < 0.05); CI: Confidence interval; β: Unstandardized estimate of fixed effect  Multivariate models were adjusted for baseline values of parenting outcomes and covariates (ethnicity, gender, income, antenatal course attendance, education level, religion, age, and number of children) | | | | |

**Table S3.** Pearson correlation coefficients between PDA use and parenting outcomes at one month postpartum among intervention group parents

| PDA usage \ Parenting outcomes | Parenting self-efficacy | Stress | Anxiety | Depression | Social support | Parent-child bonding | Parenting satisfaction |
| --- | --- | --- | --- | --- | --- | --- | --- |
| Educational materials | -0.140 | 0.084 | -0.184 | 0.038 | 0.047 | 0.034 | -0.073 |
| Chatbot questions | -0.113 | 0.096 | -0.049 | 0.186 | -0.008 | 0.269** | -0.152 |
| Mindfulness videos | -0.033 | -0.062 | -0.001 | 0.022 | 0.160 | 0.132 | -0.030 |
| Gratitude exercises | -0.004 | 0.027 | -0.003 | 0.087 | 0.073 | 0.226* | -0.062 |
| Reflection exercises | 0.003 | 0.009 | -0.001 | 0.069 | 0.068 | 0.203* | -0.034 |
| Posters made | 0.056 | 0.029 | 0.071 | 0.044 | 0.090 | 0.196 | 0.006 |
| Poster ‘likes’ given | 0.037 | 0.084 | 0.088 | 0.119 | 0.089 | 0.238* | -0.014 |
| Poster ‘likes’ received | 0.069 | -0.005 | 0.072 | 0.025 | 0.116 | 0.187 | -0.012 |
| Forum posts | -0.096 | 0.014 | -0.149 | 0.034 | -0.010 | 0.319** | -0.294** |
| ***Note:***  *p-value <0.05; **p-value <0.01 | | | | | | | |

**Table S4.** Regression estimates of PDA use on parenting outcomes at three months postpartum among intervention group parents in multivariate models

| **Variable** | **β (95%CI)** | **Standard Error (SE)** | **t-value** | **p-value** |
| --- | --- | --- | --- | --- |
| **Outcome 1: Parenting self-efficacy** | | | | |
| Number of educational materials | 0.03 (-0.19 to 0.25) | 0.11 | 0.30 | 0.763 |
| Number of chatbot questions | -0.02 (-0.15 to 0.12) | 0.07 | -0.22 | 0.826 |
| Number of mindfulness videos | -0.26 (-0.98 to 0.45) | 0.36 | -0.74 | 0.464 |
| Number of gratitude exercises | -0.94 (-6.04 to 4.15) | 2.55 | -0.37 | 0.713 |
| Number of reflection exercises | 1.14 (-3.66 to 5.94) | 2.40 | 0.48 | 0.636 |
| Number of posters made | -0.12 (-2.07 to 1.82) | 0.97 | -0.12 | 0.902 |
| Number of poster ‘likes’ given | -0.08 (-0.51 to 0.34) | 0.21 | -0.40 | 0.692 |
| Number of poster ‘likes’ received | 0.13 (-0.38 to 0.63) | 0.25 | 0.50 | 0.621 |
| Number of forum posts | -0.28 (-1.60 to 1.04) | 0.66 | -0.42 | 0.673 |
| **Outcome 2: Stress** | | | | |
| Number of educational materials | 0.18 (-0.10 to 0.46) | 0.14 | 1.30 | 0.199 |
| Number of chatbot questions | -0.07 (-0.24 to 0.09) | 0.08 | -0.89 | 0.375 |
| Number of mindfulness videos | -0.55 (-1.41 to 0.30) | 0.43 | -1.29 | 0.201 |
| Number of gratitude exercises | 3.21 (-3.00 to 9.42) | 3.11 | 1.03 | 0.306 |
| Number of reflection exercises | -2.78 (-8.60 to 3.05) | 2.92 | -0.95 | 0.344 |
| Number of posters made | -0.40 (-2.75 to 1.95) | 1.18 | -0.34 | 0.737 |
| Number of poster ‘likes’ given | 0.11 (-0.39 to 0.61) | 0.25 | 0.45 | 0.653 |
| Number of poster ‘likes’ received | 0.27 (-0.35 to 0.88) | 0.31 | 0.86 | 0.392 |
| Number of forum posts | -1.27 (-2.85 to 0.32) | 0.79 | -1.60 | 0.115 |
| **Outcome 2: Stress (Additional model built with univariate factors of p-value < 0.1)** | | | | |
| Number of educational materials | 0.07 (-0.15 to 0.28) | 0.11 | 0.61 | 0.541 |
| Number of posters made | -0.16 (-1.11 to 0.80) | 0.48 | -0.32 | 0.747 |
| Number of poster ‘likes’ given | 0.22 (-0.12 to 0.57) | 0.17 | 1.29 | 0.203 |
| **Outcome 3: Anxiety** | | | | |
| Number of educational materials | -0.18 (-0.60 to 0.24) | 0.21 | -0.86 | 0.392 |
| Number of chatbot questions | 0.06 (-0.19 to 0.32) | 0.13 | 0.50 | 0.622 |
| Number of mindfulness videos | 0.16 (-1.19 to 1.52) | 0.68 | 0.24 | 0.809 |
| Number of gratitude exercises | -0.53 (-10.4 to 9.35) | 4.94 | -0.11 | 0.916 |
| Number of reflection exercises | -1.66 (-10.87 to 7.55) | 4.61 | -0.36 | 0.721 |
| Number of posters made | 0.07 (-3.62 to 3.75) | 1.85 | 0.04 | 0.971 |
| Number of poster ‘likes’ given | -0.19 (-0.98 to 0.60) | 0.40 | -0.49 | 0.628 |
| Number of poster ‘likes’ received | 0.57 (-0.40 to 1.55) | 0.49 | 1.18 | 0.242 |
| Number of forum posts | -2.45 (-5.01 to 0.11) | 1.28 | -1.92 | 0.060 |
| **Outcome 4: Depression** | | | | |
| Number of educational materials | 0.05 (-0.13 to 0.23) | 0.09 | 0.58 | 0.567 |
| Number of chatbot questions | 0.03 (-0.08 to 0.14) | 0.05 | 0.55 | 0.587 |
| Number of mindfulness videos | -0.25 (-0.82 to 0.31) | 0.28 | -0.89 | 0.377 |
| Number of gratitude exercises | 1.58 (-2.51 to 5.66) | 2.05 | 0.77 | 0.444 |
| Number of reflection exercises | -1.77 (-5.60 to 2.07) | 1.92 | -0.92 | 0.361 |
| Number of posters made | 0.31 (-1.23 to 1.85) | 0.77 | 0.41 | 0.687 |
| Number of poster ‘likes’ given | 0.01 (-0.32 to 0.35) | 0.17 | 0.08 | 0.935 |
| Number of poster ‘likes’ received | -0.004 (-0.41 to 0.40) | 0.20 | -0.02 | 0.982 |
| Number of forum posts | -0.71 (-1.76 to 0.35) | 0.53 | -1.34 | 0.185 |
| **Outcome 5: Social support** | | | | |
| Number of educational materials | 0.09 (-0.12 to 0.31) | 0.11 | 0.86 | 0.396 |
| Number of chatbot questions | -0.02 (-0.14 to 0.12) | 0.07 | -0.23 | 0.822 |
| Number of mindfulness videos | 0.26 (-0.44 to 0.95) | 0.35 | 0.74 | 0.464 |
| Number of gratitude exercises | 2.73 (-2.32 to 7.79) | 2.53 | 1.08 | 0.285 |
| Number of reflection exercises | -3.71 (-8.45 to 1.02) | 2.37 | -1.57 | 0.122 |
| Number of posters made | 0.11 (-1.79 to 2.01) | 0.95 | 0.12 | 0.908 |
| Number of poster ‘likes’ given | -0.29 (-0.69 to 0.12) | 0.20 | -1.43 | 0.159 |
| Number of poster ‘likes’ received | 0.12 (-0.38 to 0.62) | 0.25 | 0.47 | 0.637 |
| Number of forum posts | -0.21 (-1.49 to 1.08) | 0.64 | -0.32 | 0.751 |
| **Outcome 5: Social support (Additional model built with univariate factors of p-value < 0.1)** | | | | |
| Number of gratitude exercises | 3.09 (-0.57 to 6.75) | 1.84 | 1.68 | 0.097 |
| Number of reflection exercises | -3.32 (-7.07 to 0.43) | 1.88 | -1.77 | 0.081 |
| Number of poster ‘likes’ given | -0.23 (-0.45 to -0.01) | 0.11 | -2.11 | 0.038* |
| **Outcome 6: Parent-child bonding** | | | | |
| Number of educational materials | -0.02 (-0.11 to 0.07) | 0.04 | -0.54 | 0.593 |
| Number of chatbot questions | -0.001 (-0.05 to 0.05) | 0.03 | -0.02 | 0.984 |
| Number of mindfulness videos | -0.007 (-0.29 to 0.28) | 0.14 | -0.05 | 0.962 |
| Number of gratitude exercises | -0.56 (-2.61 to 1.49) | 1.03 | -0.55 | 0.585 |
| Number of reflection exercises | 0.75 (-1.17 to 2.68) | 0.96 | 0.78 | 0.438 |
| Number of posters made | 0.57 (-0.20 to 1.34) | 0.39 | 1.47 | 0.146 |
| Number of poster ‘likes’ given | -0.02 (-0.18 to 0.15) | 0.08 | -0.23 | 0.817 |
| Number of poster ‘likes’ received | -0.17 (-0.37 to 0.04) | 0.10 | -1.64 | 0.107 |
| Number of forum posts | -0.21 (-0.76 to 0.33) | 0.27 | -0.78 | 0.436 |
| **Outcome 7: Parenting satisfaction** | | | | |
| Number of educational materials | 0.06 (-0.38 to 0.50) | 0.22 | 0.26 | 0.792 |
| Number of chatbot questions | 0.06 (-0.21 to 0.32) | 0.13 | 0.42 | 0.675 |
| Number of mindfulness videos | 0.95 (-0.46 to 2.36) | 0.70 | 1.35 | 0.182 |
| Number of gratitude exercises | 0.70 (-9.49 to 10.88) | 5.10 | 0.14 | 0.892 |
| Number of reflection exercises | -1.72 (-11.28 to 7.84) | 4.79 | -0.36 | 0.721 |
| Number of posters made | -0.36 (-4.28 to 3.56) | 1.96 | -0.18 | 0.856 |
| Number of poster ‘likes’ given | -0.69 (-1.52 to 0.14) | 0.42 | -1.66 | 0.102 |
| Number of poster ‘likes’ received | 0.34 (-0.67 to 1.35) | 0.51 | 0.68 | 0.500 |
| Number of forum posts | 0.21 (-2.51 to 2.92) | 1.36 | 0.15 | 0.879 |
| **Outcome 7: Parenting satisfaction (Additional model built with univariate factors of p-value < 0.1)** | | | | |
| Number of educational materials | 0.31 (0.01 to 0.61) | 0.15 | 2.07 | 0.042* |
| Number of poster ‘likes’ given | -0.50 (-0.84 to -0.16) | 0.17 | -2.93 | 0.005* |
| Number of forum posts | 1.19 (-0.82 to 3.21) | 1.01 | 1.18 | 0.241 |
| ***Note:***  *significant result (p-value < 0.05); CI: Confidence interval; β: Unstandardized estimate of fixed effect  Multivariate models were adjusted for baseline values of parenting outcomes and covariates (ethnicity, gender, income, antenatal course attendance, education level, religion, age, and number of children) | | | | |

**Table S5.** Pearson correlation coefficients between PDA use and parenting outcomes at three months postpartum among intervention group parents

| PDA usage \ Parenting outcomes | Parenting self-efficacy | Stress | Anxiety | Depression | Social support | Parent-child bonding | Parenting satisfaction |
| --- | --- | --- | --- | --- | --- | --- | --- |
| Educational materials | -0.059 | 0.081 | -0.141 | 0.029 | -0.106 | -0.022 | 0.010 |
| Chatbot questions | -0.021 | 0.116 | -0.065 | 0.099 | -0.142 | 0.001 | 0.003 |
| Mindfulness videos | 0.023 | <0.0001 | 0.009 | -0.033 | -0.063 | -0.007 | 0.049 |
| Gratitude exercises | 0.041 | 0.116 | -0.045 | 0.029 | -0.166 | -0.009 | 0.042 |
| Reflection exercises | 0.048 | 0.091 | -0.038 | 0.002 | -0.166 | -0.012 | 0.054 |
| Posters made | 0.107 | 0.135 | 0.091 | 0.068 | -0.095 | -0.020 | 0.081 |
| Poster ‘likes’ given | 0.032 | 0.201* | 0.099 | 0.094 | -0.134 | 0.047 | -0.006 |
| Poster ‘likes’ received | 0.114 | 0.102 | 0.073 | 0.040 | -0.069 | -0.051 | 0.093 |
| Forum posts | -0.042 | -0.006 | -0.211* | -0.056 | -0.016 | -0.025 | -0.024 |
| ***Note:***  *p-value <0.05 | | | | | | | |

**Table S6.** Time taken to complete surveys

| **Variable** | **Mean (SD)** | **Range (min-max)** |
| --- | --- | --- |
| Time taken to complete baseline survey  (days) | 1.22 (4.74) | 0-38 |
| Time taken to complete one-month postpartum survey (days) | 6.58 (8.86) | 0-35 |
| Time taken to complete three-months postpartum survey (days) | 5.05 (8.16) | 0-37 |

**Table S7.** Significant sociodemographic variables affecting time taken to complete PDA study surveys among all parents in multivariate model

| **Variable** | **Mean (Standard deviation)** | **MD (95%CI) or β (95%CI)** | **F-value** | **P-value** |
| --- | --- | --- | --- | --- |
| **Time taken to complete baseline survey (days)** | | | | |
| Age |  | -0.15 (-0.28 to -0.021) | 5.21 | 0.024 |
| Ethnicity |  |  | 6.82 | <0.001 |
| Chinese | 0.26 (0.82) | -0.47 (-1.89 to 0.94) |  | 0.510 |
| Malay | 0.10 (0.30) | -0.93 (-3.10 to 1.23) |  | 0.396 |
| Indian | 0.82 (1.43) | 3.48 (0.92 to 6.05) |  | 0.008 |
| Others* | 1.11 (1.83) |  |  |  |
| **Time taken to complete one month postpartum survey (days)** | | | | |
| Grouping |  |  | 7.79 | 0.006 |
| Intervention | 8.35 (9.78) | 3.51 (1.03 to 5.99) |  |  |
| Control* | 4.28 (6.90) |  |  |  |
| **Time taken to complete three months postpartum survey (days)** | | | | |
| Number of children |  | 1.94 (0.55 to 3.33) | 7.59 | 0.007 |
| Ethnicity |  |  | 5.06 | 0.002 |
| Chinese | 3.77 (6.40) | -2.89 (-6.36 to 0.58) |  | 0.102 |
| Malay | 3.37 (5.18) | -9.72 (-15.06 to -4.37) |  | <0.001 |
| Indian | 6.24 (9.58) | -3.88 (-10.55 to 2.79) |  | 0.253 |
| Others* | 7.07 (9.33) |  |  |  |
| ***Note:***  *Reference group; CI: Confidence interval; MD: Mean difference; β: Unstandardized estimate of fixed effect  Multivariate models are adjusted for the following variables: grouping, ethnicity, gender, income, antenatal course attendance, education level, religion, age, and number of children. All MD (95%CI)s are calculated based on the estimated marginal means. | | | | |
